# IFIM: a database of integrated fitness information for microbial genes

**DOI:** 10.1093/database/bau052

**Published:** 2014-06-11

**Authors:** Wen Wei, Yuan-Nong Ye, Sen Luo, Yan-Yan Deng, Dan Lin, Feng-Biao Guo

**Affiliations:** Center of Bioinformatics and Key Laboratory for NeuroInformation of the Ministry of Education, School of Life Science and Technology, University of Electronic Science and Technology of China, Chengdu 610054, China

## Abstract

Knowledge of an organism’s fitness for survival is important for a complete understanding of microbial genetics and effective drug design. Current essential gene databases provide only binary essentiality data from genome-wide experiments. We therefore developed a new database that Integrates quantitative Fitness Information for Microbial genes (IFIM). The IFIM database currently contains data from 16 experiments and 2186 theoretical predictions. The highly significant correlation between the experiment-derived fitness data and our computational simulations demonstrated that the computer-generated predictions were often as reliable as the experimental data. The data in IFIM can be accessed easily, and the interface allows users to browse through the gene fitness information that it contains. IFIM is the first resource that allows easy access to fitness data of microbial genes. We believe this database will contribute to a better understanding of microbial genetics and will be useful in designing drugs to resist microbial pathogens, especially when experimental data are unavailable.

**Database URL:** http://cefg.uestc.edu.cn/ifim/ or http://cefg.cn/ifim/

## Introduction

Essential genes are genes that are considered to be ‘essential’ for the survival of an organism (1, 2). The genome-wide identification of essential genes has been performed by single-gene knockout (3, 4), transposon mutagenesis (5, 6) and RNA interference (7). Because the experimental techniques are challenging and time-consuming, computational methods offer an appealing alternative for predicting essential genes without the need for expensive and difficult screening.

Based on the hypothesis that essential genes are persistent during the long evolutionary process, we recently developed a new prediction tool (Geptop) that offers gene essentiality annotations for bacterial genomes (8) (http://cefg.uestc.edu.cn/geptop/). Although the Geptop method uses a training process that is similar to a previously reported algorithm (9), it differs in that bacterial essential genes are predicted using only evolutionary features of the genomes, which significantly improved its performance compared with other existing methods. Most of the cross-organism predictions yielded AUC (area under the receiver operating characteristic curve) scores over 0.8, especially 0.98 for the profiling of the *Escherichia coli* chromosome data set (http://www.shigen.nig.ac.jp/ecoli/pec/). The Geptop tool can effectively predict essential genes in every sequenced bacterial genome, and it has been estimated that most of these predictions will yield AUC scores that exceed 0.7.

Essential bacterial gene products have often proved to be attractive drug targets in the development of antibiotics (10, 11). The study of on an organism’s fitness for survival is an important step towards understanding microbial genetics. Previous studies have reported a negative correlation between essential function and the rate of coding sequence evolution (12–14). Genome-wide gene fitness data have been found to be more useful than binary data of essential or nonessential genes in some studies; for example, for inferring gene regulatory networks (15), identifying potential virulence genes and drug targets (16–18), revealing different fitness actions in distinct environments (19, 20) and directly detecting conditional essential genes. However, current essential gene databases provide only binary essentiality data from genome-wide experiments (21, 22), and different fitness among genes has not been the focus of these databases. We therefore developed a new database named Integrated Fitness Information for Microbial genes (IFIM, http://cefg.uestc.edu.cn/ifim/ or http://cefg.cn/ifim/).

## Construction and Content of the IFIM Database

**Figure 1.**
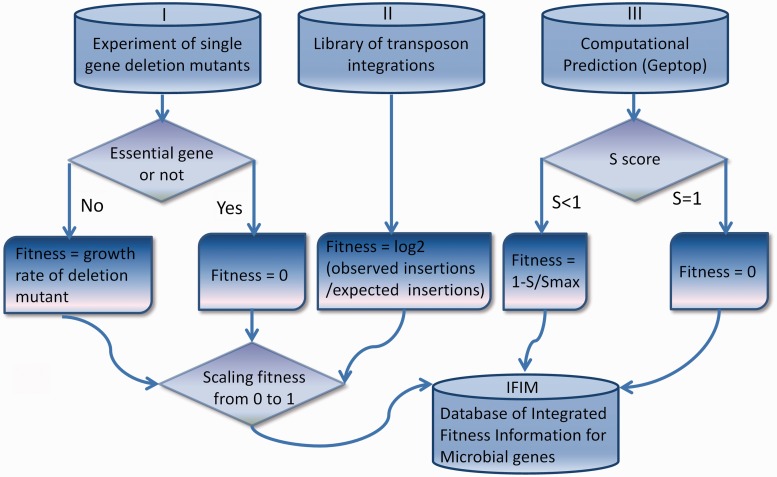
Details of data collection and processing for the IFIM database.

The integrated fitness data in IFIM originate from experiments of single-gene deletion mutants, libraries of transposon integrations and computational simulations using Geptop. Details of data collection and processing are described in this section and shown in Figure 1.

### Collection of Fitness Data From Experimental Data

In single-gene deletion mutant experiments, the contribution of a gene to fitness is usually measured as the growth rate of its deletion mutant (23). We assigned essential genes a deletion mutant growth rate of zero (Figure 1). For transposon integration libraries, the fitness of a gene was defined as the degree to which the gene tolerated transposon insertions (Figure 1) (24). We collected all microbial data of transposon integrations and single-gene deletion mutants that were currently available to compute fitness. Some of these data sources (SC02 of *Saccharomyces cerevisiae*, EC02 of *E. coli*, STM01 of *Salmonella typhimurium* 14028S, STY01 of *S. typhimurium* LT2 and NM01 of *Neisseria meningitides* MC58) have not been included in two well-known essential gene databases: the Database of Essential Genes (DEG) (21) and the Online GEne Essentiality database (OGEE) (22). The yeast genome annotation was downloaded from the Saccharomyces Genome Database (25), and the annotations for the other species were obtained from GenBank (26). We updated the gene information for the mutation experiments in the original data sources to make it consistent with the current annotations. The fitness data were scaled from 0 to 1 in each of the genomes.

### Prediction of Fitness by Computational Simulation

For most of bacterial species, the deletion/insertion mutant experimental data were not available. Therefore, we used Geptop, which can effectively predict essential genes under various nutritional conditions, as an alternative to genome-wide fitness data. We performed computational simulations for all the sequenced bacterial genomes that are currently available in GenBank. To a certain genome, we first generated a proteome by translating all the protein coding sequences in the genome, and then, used Geptop to predict an essentiality score (S) for each gene. The fitness value for a gene was defined as 0 when S was equal to 1. When S was not equal to 1, we defined fitness as 1-S/Smax, where Smax is the maximum S (excluding S = 1) in the genome.

**Figure 2.**
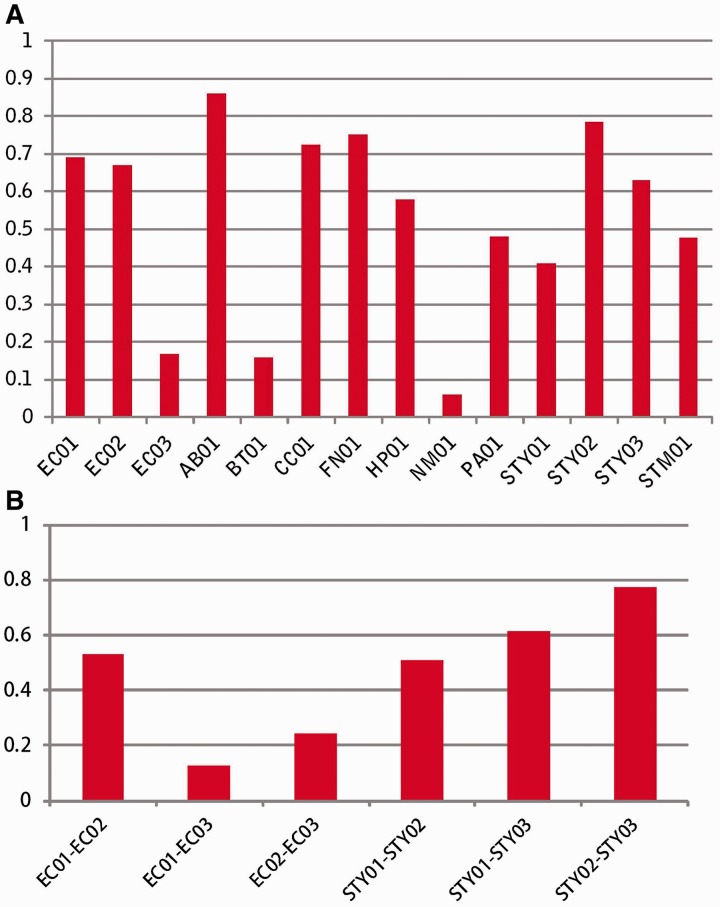
Assessment of the accuracy of prediction. (**A**) Linear regression *R*^2^ between experiment-derived fitness and computation-predicting fitness, (**B**) Correlation analysis among experimental fitness data sets.

To assess the accuracy of the fitness prediction, we performed a linear regression (*R*^2^) analysis between experimentally derived fitness data sets and the computationally predicted fitness (Figure 2A). Most *R*^2^ values were >0.45, which indicated that gene fitness was predicted well using this method. *R*^2^ values were also used in a correlation analyses among three experimental fitness data sets of *E. coli* and among three experiment-based data sets of *S. typhimurium* LT2 (Figure 2B). We found that the correlations between the experimental data and predicted data were similar to correlations among these experimental fitness data sets. Therefore, when experimental fitness data are not available, the predicted fitness value can be taken as an alternative measure.

**Figure 3.**
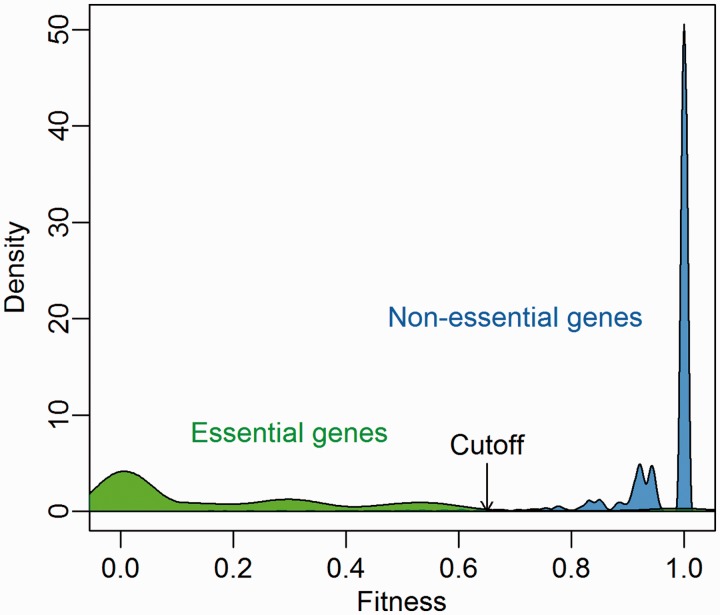
Predicted fitness distribution of essential and nonessential genes in 21 bacterial strains. Essentiality information was obtained from the DEG database.

We extracted gene essentiality information of 21 bacterial strains from the DEG database (version 7.5) and investigated the predicted fitness distribution of essential and nonessential genes. As Figure 3 shows, there is a clear gap from 0.65 to 0.75 between essential genes and nonessential genes, which corresponds to the Geptop default cutoff point. Therefore, we recommend a cutoff of 0.65 is used to separate essential and nonessential genes based on Geptop predicted fitness.

**Figure 4.**
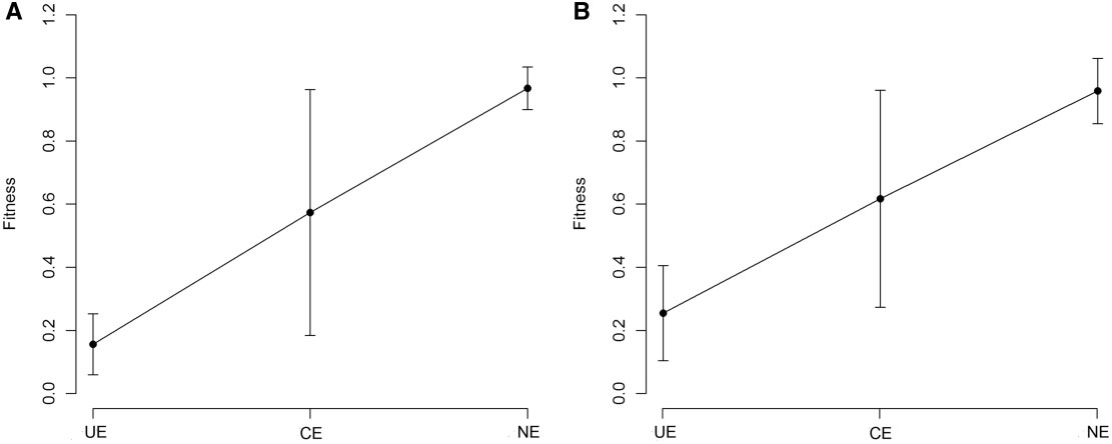
Geptop predicted fitness values. (**A**) Predicted fitness values for *E. coli*. (**B**) Predicted fitness values for *S. typhimurium*. Error bars represent 90% confidence intervals on the estimates of the means. UE: universal essential; CE: conditional essential; NE: nonessential.

One of the advantages of OGEE is the inclusion of the concept of conditional essentiality (22). Most of reference sets in Geptop follow experiment settings under favorable environmental condition. Therefore, the Geptop predictions could be a result with optimal conditions. The concept of conditional essentiality could explain why Geptop results correlated well with the EC01 and EC02 data sets, but not so well with the EC03 data set. The minimal bacterial gene set has been estimated to contain 250–300 gene candidates (27). We extracted ∼300 essential genes from the EC01, EC02, EC03, STY01, STY02 and STY03 data sets based on the lowest experimental fitness values, respectively. Following the concept of conditional essentiality, we identified the intersection among the EC01, EC02 and EC03 data sets (and the STY01, STY02 and STY03 data sets) as the universal essential (UE) genes for *E. coli* (*S. typhimurium*), whereas the other essential genes were identified as conditional essential (CE) genes. As Figure 4 shows, the UE genes have smaller predicted fitness values (most were between 0.1 and 0.3), indicating that the UE genes are of major importance so they were identified as essential genes regardless of the experimental settings. CE genes are a set of selective essential genes that may be essential genes under some experimental conditions but not under other conditions; so CE genes should have medium fitness values. The means of the predicted fitness values for the *E. coli* and *S. typhimurium* data sets for the CE genes were ∼0.55 for both data sets (Figure 4), which is close to the prediction gap (0.65–0.75) between the essential genes and nonessential genes (Figure 3). Therefore, another advantage of IFIM is that it might provide the possibility of distinguishing between putative UE and CE genes using a rough cutoff of ∼0.3.

Gene essentiality can be disrupted by duplication, even in closely related species. The more recent the duplication event, the more likely it will be that both duplicates are not essential (28). We used all 296 single-copy essential genes of *E. coli*, which are used as a reference set in Geptop, to test the impact of duplication events on gene essentiality. Similar to the Cluster of Essential Genes database (CEG) (29), in IFIM, only the gene name is used to identify multiple copy genes. By searching the annotations of *Salmonella enterica*, we found that the *E. coli* essential genes *ssb*, *ftsl* and *trpS* had multiple copies in *S. enterica* serovar Typhi Ty2, and *trpS* had multiple copies in *S. enterica* serovar Typhimurium 14028S. Comparisons of the fitness values among the computational predictions and experiment data from the STY01, STY02, STY03 and STM01 data sets (Table 1) showed that the Geptop predictions for *ssb* and *ftsl* were in complete agreement with the experimental data; namely, one copy of each of the genes was an essential gene but the other copy was not. However, we found a contradiction between the computational prediction and experiment data for *trpS*. One copy of *trpS* had a relatively low fitness value based on Geptop result and was therefore predicted to be an essential gene, whereas the other copy of *trpS* was predicted to be a nonessential gene. However, the experimental data showed that the two copies of *trpS* had high fitness values and tended to be nonessential. It is possible that the *trpS* gene duplicated recently and this event disrupted the gene essentiality. This finding may reflect a limitation of Geptop to predict essentiality for recently duplicated genes. However, compared with eukaryotic genomes, gene duplication occurs only rarely in bacterial genomes. According to our statistics, only ∼1% of the *E. coli* essential genes have multiple copies in *S. enterica*.

**Table 1.** Fitness values of multiple copy genes in the computational predictions and experiment data from four data sets

*S. enterica* serovar typhi Ty2				
Gene	Dataset			
	Geptop	STY01	STY02	STY03
*Ssb*	t4161 (0.237)	t4161 (0.231)	t4161 (0.245)	t4161 (0.260)
	t4237 (0.733)	t4237 (0.742)	t4237 (0.837)	t4237 (0.833)
*ftsI*	t0126 (0.263)	t0126 (0.102)	t0126 (0.128)	t0126 (0.142)
	t1042 (0.835)	t1042 (0.616)	t1042 (0.780)	t1042 (0.772)
*trpS*	t4024 (0.448)	t4024 (0.419)	t4024 (0.863)	t4024 (0.563)
	t4557 (0.834)	t4557 (0.559)	t4557 (0.819)	t4557 (0.689)

*S. enterica* serovar typhimurium 14028S		
	Geptop	STM01
*trpS*	STM14_4193 (0.405)	STM14_4193 (0.694)
	STM14_5412 (0.821)	STM14_5412 (0.764)

### Statistics

The IFIM database currently covers 16 genome-wide experimental identifications in 11 organisms and contains records of the theoretical predictions for 2186 bacterial genomes. IFIM overlaps with the existing DEG, OGEE and CEG databases, but differs from them in that it provides quantitative fitness values for the genes.

## Utility

The web interface for the IFIM database is freely available at http://cefg.uestc.edu.cn/ifim/. The interface provides links to four pages: Home & Browse, Analysis, Download and Links.

**Figure 5.**
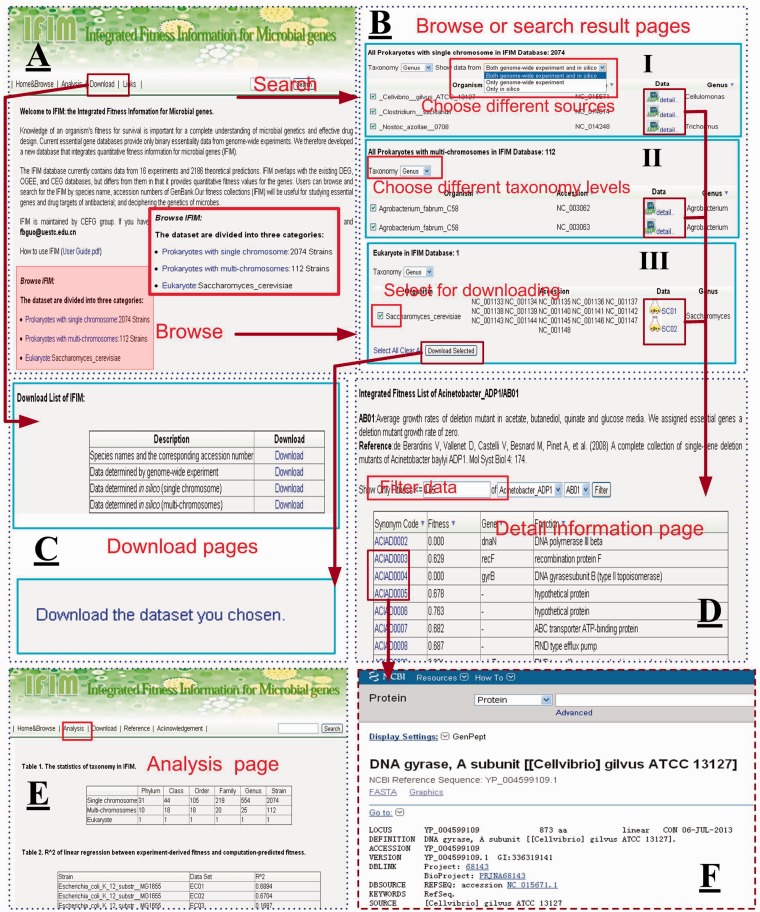
The web interface for the IFIM database. (**A**) The Home & Browse page; (**B**) Examples of Browse or Search result pages. (I) choosing different sources to browse data sets, (II) choosing different taxonomy levels to browse data sets and (III) choosing different data sets to download; (**C**) The Download pages; (**D**) An example of a fitness information page; (**E**) The Analysis page; (**F**) The link page showing a NCBI link to a gene.

The Home & Browse module is the default page that is displayed when users access the IFIM database. The page is in two parts (Figure 5A). In the top part of the page, a brief introduction to IFIM and a link to the user guide are provided. Below this is a list of the three data sets that are available: prokaryotes with single chromosome, prokaryotes with multi-chromosomes and eukaryote, which is *S. cerevisiae*. Users can click on the links to browse the individual data sets (Figure 5B). For each data set, a table is displayed that lists the contents of the data set based on the ascending alphabet order of ‘organism’. Users can reform the table by choosing from different taxonomy levels (phylum, class, order, family and genus) that are shown in a dropdown list at the top of the table (Figure 5B, II); by default, the strains are sorted by genus.

Several of the strains in the prokaryote with single chromosome data set have both experimental and computational data associated with them. Different icons are used to distinguish between the experimental and computational data. By clicking on these icons, users can browse gene fitness information. Alternatively, users can select the most appropriate data using a dropdown list at the top of the table (Figure 5B, I) that allows users to choose both genome-wide and *in silico*, only genome-wide or only *in silico* data. On the gene fitness page, a table that lists synonym code, fitness value, gene name and gene function of each gene in the selected species is given. All genes are presented by default, but users can set the fitness value to filter the list of genes (Figure 5D). We recommend using a cutoff of 0.65 to distinguish between Geptop predicted essential and nonessential genes. The listed data can also be displayed in ascending or descending order by clicking the column name in the header. For experimental data sets, the experimental setting and reference are also given. The synonym code is hyperlinked to the NCBI database so that users can retrieve detailed information for each gene (Figure 5F).

At the top of each page in the IFIM interface, there is a Search module that users can use to search the IFIM database by organism name or accession number (Figure 5A). Users can use the search box from any page to rapidly ocate information of interest. The Search module supports fuzzy queries of either organism name or accession number. For example, users can input ‘Escherichia_coli’ or ‘Escherichia coli’ in the search box to find all strains of *E. coli* in the IFIM database. Temporarily, the abbreviation ‘E. coli’ is not supported. The results page for a search is similar to the page that is displayed from the Browse module when a user clicks on a link to one of the three kinds of data sets (Figure 5B).

The Download module provides two ways to download data sets. Users can either download all the data sets from the Download page (Figure 5C, I) or they can choose data sets from the pages displayed from the Browse page or from a Search result page to download (Figure 5B, III).

The Analysis page presents a basic statistical analysis of the information in the database. The Links page provides links to related web resources and servers.

## Future Directions

Although other essential gene databases such as DEG and OGEE are widely used, IFIM is the only database that provides integrated microbial fitness data from both experiments and computational simulations. The IFIM database currently contains data from 16 experiments and 2186 theoretical predictions. The quantitative fitness data may be more useful than binary data for studies into essential and nonessential gene.

The numbers of essentiality experiments that are being reported are continually increasing; therefore, we will continue to update IFIM to cover more experiments and more species, as well as integrate more information about each IFIM data set. We will also continue to develop the Geptop software to help increase the accuracy of predicting fitness. We believe the IFIM database will contribute to a better understanding of microbial genetics and will also be useful in drug design. Special attention will also be paid to detecting CE genes, identifying potential virulence genes and drug targets, inferring gene regulatory networks and revealing different fitness actions in distinct environments.
